# Violence, abuse and discrimination: key factors militating against control of HIV/AIDS among the LGBTI sector

**DOI:** 10.1080/17290376.2018.1492960

**Published:** 2018-07-19

**Authors:** Dominic Targema Abaver, Elphina Nomabandla Cishe

**Affiliations:** a Division of Academic Affairs and Research, Directorate of Research, Innovation and Development, Walter Sisulu University, NMD, Mthatha, South Africa; b Directorate of Research Innovation and Development, Walter Sisulu University, Nelson Mandela Drive, Mthatha, Eastern Cape

**Keywords:** Social stigma, LGBTI, students, HIV/AIDS

## Abstract

The Lesbian, Gay, Bisexual, Transgender and Intersex (LGBTI) South Africans continue to face considerable challenges, including societal stigma, homophobic violence (particularly corrective rape), and high rates of sexually transmitted diseases and infections (particularly Human Immunodeficiency Virus (HIV)/AIDS) even when discrimination based on sexual orientation was outlawed by South African’s post-apartheid constitution. This study was conducted to ascertain violence, abuse and discrimination against the LGBTI sector as key factors that hinder the smooth implementation of HIV/AIDS programme among sexually minority (LGBTI) group in Walter Sisulu University, South Africa. The self-structured questionnaire was used to collect data. The study involved 3048 purposively selected participants (1285 male and 1763 female) aged 17–38 years. About 70.5% of the participants witnessed physical attack as a form of violence against people in same-gender relationship; 47.7% disagreed that violent targeted at this sexually minority group is justified. The LGBTI face challenges which include verbal insults (937, 32.4%), bullying (532, 18.4%) and name-calling (1389, 48%). Discrimination against members of the LGBTI sector was witnessed in various forms: non-acceptance (981, 33.9%), disapproval of act of homosexuals (1308, 45.2) and denial of rights (327, 11.3). Violence, abuse and discrimination which constitute stigmatisation among the LGBTI sector are received with mix feeling. Some respondents justified the use of one or more of these key elements of stigmatisation against the LGBTI (6.6%, supports violence), others condemned these acts of stigmatisation (28.8%), against discrimination). Social stigma which resulted from violence, abuse and discrimination exist in this institution and is responsible for the unwillingness of disclosure of sexual orientation among the LGBTI members. An enabling environment should be created where the LGBTI members could come out freely to access programmes targeted at the prevention and control of HIV/AIDS.

## Introduction

The Lesbian, Gay, Bisexual, Transgender and Intersex (LGBTI) are a group of people who share some things in common, and within which there is also diversity. Communities with intersect and overlap do exist within the group, which makes it difficult to make reference to the group as a single or unified ‘LGBTI community’. It is worthy of note that people within these communities may or may not be involved publicly with ‘LGBTI’ organisations; may or may not know anyone else who is ‘LGBTI’; may or may not feel welcome or comfortable attending ‘LGBTI’ events/services; may or may not live as and identify as heterosexual. However, for the purpose of this study the term ‘LGBTI community’ is used to describe this group of students at Walter Sisulu University and other groups operating within and outside South Africa, whose members share common characteristics regarding sexual orientation. The theoretical framework used in describing various indices of stigmatisation in this study followed the oppression and social justice approach described by Hardiman and Jackson (1997). These authors (Hardiman & Jackson, 1997) identify four conditions for the existence of social oppression: (1) a ‘one up group’ that has the power to define and determine reality, normality and correctness; (2) ‘systemic and institutionalized discrimination, harassment, exploitation, marginalization and other forms of differential treatment’; (3) psychological colonisation of the ‘one down group’ through socialising them to internalise their oppressed condition and collude with the oppressors’ ideology and social system and (4) misrepresentation, discounting or even eradication of the oppressed culture, language and history, while imposing the culture of the dominant groups.

A stigmatised person, according to Crocker, Major, and Steele (1998), has an attribute that conveys a devalued social identity within a particular context. It is this devaluation that leads to a variety of events that result in a stigmatised person exceeding his or her adaptive resources (Lazarus & Folkman, 1984). Miller and Kaiser (2001, p. 73) state that stigmatised people have a vast array of responses to stressors resulting from devalued social status, including emotional, cognitive, biological and behavioural responses. Though the LGBTI populations are covered by the Universal Declaration of Human Rights of 1948, the document is based on the principle that the rights articulated are fundamental to all human beings and that these are derived from shared human dignity (United Nations, 2016). Though sexual and gender minority rights are not included in the specific language of the text, the assertion by the first article that ‘All human beings are born free and equal in dignity and rights’ implies that members of the LGBTI community are protected by the document. Despite this, the LGBTI community members globally face discrimination in all facets of life.

Violence, abuse and discrimination have been associated with homosexuality. Some members of LGBTI community have been victims of pervasive violent abuse, harassment, discrimination and hate-related killings in all regions of the world. The United Nation’s report of 2015 elaborates on a Geneva-based Human Rights Council report which cites several cases of violence against members of the LGBTI community. These include the murder of transsexual women in Uruguay and of black lesbian women in South Africa, and the killing of a gay man in Chile by neo-Nazis who carved *swastikas* into his body (United Nation Report, 2015). The report further states that, in February 2015, photos appeared to show several men, allegedly accused of homosexual acts, being pushed off a building to their deaths in Syria by militants of the so-called Islamic State extremist group*.* Other instances of such violence as recorded in the UN (2015) report include: a woman who was reportedly arrested in Bangladesh for being a lesbian and was allegedly raped by police while in custody; and four people arrested in Egypt for their alleged sexual orientation who were reportedly sexually assaulted by other inmates while in detention (United Nations Report, 2015). The report of the Geneva-based Human Rights Council concludes that, at least 76 countries retain laws used to criminalise and harass people on the basis of their sexual orientation and gender identity or expression, including laws criminalising consensual same-sex relationships among adults.

In a related development, a trans-murder monitoring project in Brazil, which collects reports of homicides of transgender people, lists 1612 murders in 62 countries between 2008 and 2014 (UN report, 2015).

The inter-American commission on human rights (2015) reported 594 hate-related killings of LGBTI people in the 25 countries of the Organisation of American States between January 2013 and March 2014. In Zimbabwe, many examples of non-lethal violence including arrest, beating and ill-treatment by police are witnessed. Most African countries criminalised same-sex relationship and considered the act as taboo (Mudavanhu, 2010), though Kenya and South Africa have constitutions that protect persons from exposure to stigma and discrimination based on sex (Ellis, 2009; Human Rights Watch, 2011). Mavhandu-Mudzusi (2017) reports about open condemnation of homosexuality by senior politicians and religious leaders in Zimbabwe, and people with such sexual orientation often suffer violence and aggression (Mabvurira, Motsi, Masuka, & Chigondo, 2012; Smith, 2015). The LGBTI community in South Africa faces considerable challenges which include social stigma, homophobic violence (particularly corrective rape) and high rates of HIV/AIDS infections, notwithstanding the South African constitution which outlawed discrimination based on sexual orientation, and legalised same-sex marriage (South African Government, 1996).While researching on ‘Impact of stigma and discrimination on sexual wellbeing of LGBTI students in a South African rural university’, Mavhandu-Mudzusi (2017) observed that LGBTI students at this university engage in diverse sexual practices. This is an attempt to be ‘seen as straight’ in order to avert stigma and discrimination. Experiences of violence and victimisation are frequent for LGBTI individuals and have long-lasting effects on the individual and the community (Roberts, Austin, & Corliss, 2010). Other issues encountered from an early age by the LGBTI people include being targeted for bullying, assault and discrimination which lead to depression and other mental health issues in adulthood (Rivers, 2001, 2004). These unfavourable treatments meted to members of the LGBTI lead to social desirability bias that often limits the disclosure of homosexuality which is viewed by many as negative attitudes (Dowling, Rodger, & Cumming, 2007). Social stigma does not only lead many LGBTI individuals to hide their status and relatively few make their orientation known to their health care providers (Hinchliff, Gott, & Galena, 2005), it also denies members of this sexually minority group the opportunity of a deserved tailored HIV interventions and better health outcomes. Though HIV transmission in South Africa is mainly by the heterosexual act, the need to target key populations such as the LGBTI community and include them on the national agenda of HIV programming cannot be overemphasised.

The emergence of LGBTI community across Africa in 2012, as noted by Beyrer (2012), occurred in the context of HIV epidemic. Indeed, the first real recognition that MSM populations have been present at all has been through HIV prevalence studies among these men (Baral et al., 2009). The issue of HIV has been a concern for LGBTI populations in South Africa since the first HIV cases in the country were identified among gay men decades ago. There are limited data about health issues of LGBTI community, because epidemiological studies often do not incorporate sexuality as a factor in data collection (Meads, Pennant, McManus, & Bayliss, 2009). Where sexual orientation and gender identity questions are not asked on surveys, it becomes imperatively difficult to estimate the number of LGBTI individuals and their health needs. However, the LGBTI community members have unique health care needs which come in various forms (King & Nazareth, 2006).

While it is proven that there is difficulty in establishing exact number of LGBTI members globally (due to the challenges stated above), the few available data on HIV bio-behavioural surveys globally suggest that there is increasingly high HIV prevalence rate among member of LGBTI community, given that sexual transmission, especially male-to-male homosexual transmission, has become the major mode of HIV transmission in recent years (Zhang et al., 2013). Members of the LGBTI community stand the risk of Human Immunodeficiency Virus (HIV) infection due to risk factors such as needle sharing and substance abuse, high-risk sexual behaviours, commercial sex work, health care access, lack of knowledge regarding HIV transmission, violence, abuse and discrimination, and mental health issues. Okal et al. (2013) comment about MSM as an integral part of an ‘at risk group’ of HIV/AIDS which include women, youth, intravenous drug users, commercial sex workers, prisoners and refugees. The MSM community is, therefore, considered a bridge to HIV infection in the general population (Ainswoth, Beyrer, & Soucat, 2003). Low- and middle-income countries bear the blunt of HIV/AIDS prevalence and incidence been contributed by members of the LGBTI community. The MSM are said to be approximately 19 times at higher risk of infection than in general population (AMFAR, 2008). Despite various applications of HIV/AIDS prevention strategies to the MSM within the LGBTI community, the high-risk sexual behaviours among this population are said to be increasing (Elford & Hart, 2003). Stigma and social discrimination is one of the main issues that influences HIV/AIDS transmission in MSM population (MSMGF, 2010). In China, a national report revealed that the proportion of newly diagnosed HIV cases due to Men having Sex with Men (MSM) contact has increased from 12.2% in 2007 to 32.5% in 2009 (State Council AIDS Working Committee of China, 2010). The Chinese national HIV prevalence rate among Men who have Sex with Men (MSM) had a 4.5-fold increase in the past 10 years: from 1.4% in 2001 to 6.3% in 2011 (Chow, Wilson, Zhang, Jing, & Zhang, 2011), yet it is still relatively low compared to other Asian countries such as Cambodia (7.8%), Indonesia (9.0%) and Thailand (24.6%) (Baral, Sifakis, Cleghorn, Beyrer, & Kalichman, 2007).

The need to intensify research on prevalence and incidence of HIV among the LGBTI community is borne from the fact that HIV/AIDS first emerged in the early 1980s among populations of gay men and other men who have sex with men (MSM) in Western Europe, North America and Australia. This reality shaped early responses to the epidemic and has had a lasting impact on the stigma associated with HIV/AIDS (Beyrer et al., 2010). A further research in this direction, targeting the LGBTI community goes a long way in accentuating the disease burden as well as establishing the link between homosexuality (as a risk factor) and HIV transmission.

In South Africa, lack of adequate data about the prevalence of HIV among members of the LGBTI community is paralleled by a lack of understanding of the cultural, structural, interpersonal and individual factors that affect protective practices in same-sex sexual activities. While several studies on same-sex sexuality and HIV/AIDS in Africa have recently been published, South Africa still has to catch up on this topic. However, study conducted by Sandfort, Nel, Rich, Reddy, and Yi (2008) in the three provinces of Gauteng, KwaZulu-Natal and Western Cape reveals that, of the 728 MSM who had ever been tested, 14.1% (*n* = 103; 9.9% of the total sample) reported being HIV-positive. The good news here is that, there are signs that the situation in South Africa is changing. The mention and inclusion of MSM in South Africa’s latest strategic plan for HIV/AIDS (South African National AIDS Council HIV and AIDS and STI Strategic Plan for South Africa, 2011) is seen as a welcome development. The plan acknowledges the lack of knowledge about the HIV epidemic among MSM, and that MSM have also not been considered to any great extent in national HIV and AIDS interventions.

Research into sexual orientation and gender identity at South Africa’s university is becoming more common. Such research explores the students’ perception of same-sex relationship and how negative the relationship impacts on the health of the community. Similar studies are aimed among many things, to advance scientific evidence and generate knowledge that can be utilised in many folds: help universities protect sexual minorities (LGBTI) and health care providers to articulate health care services that specifically meant for these sexual minorities.

A national study conducted by Higher Education AIDS (2009) which involved all higher institutions of learning, reported a 6.4% HIV prevalence rate among Eastern Cape institutions of higher learning (Twaise, Dana, Abaver, & Goon, 2014). Twaise et al. (2014), in their study, referred to the results of the national study conducted by HEAIDS in 2009 which indicate that Walter Sisulu University (in Eastern Cape) had a 13.3% HIV prevalence rate among its students, and that Eastern Cape has the highest percentage (41%) of HIV-positive students. An exploration to ascertain factors responsible for stigmatisation which limits the desirability of members of the LGBTI community to disclose their sexual orientation forms the rationale for this study. This study, therefore, was designed to ascertain violence, abuse and discrimination against the LGBTI community in Walter Sisulu University in Eastern Cape, as factors militating against smooth implementation of HIV/AIDS programme among this sexual minority group. The study aimed to seek an intervention from the school authority with regard to arresting all forms of violence, abuse and discrimination against members of the LGBTI community at this institution, which in turn provides an all-inclusive conducive learning atmosphere. With a very conducive atmosphere, members of the LGBTI community would feel free to disclose their sexual orientation, especially to their healthcare providers and stand to benefit immensely from the special medical care tailored for the group. The larger community also benefits when the health issues of the LGBTI community is properly addressed, given that the group forms an integral part of the larger community. It is hoped that this study will be able to address several issues surrounding the LGBTI community. These issues include the need to accept members of the LGBTI community in our society and avoid acts that would illicit stigmatisation. This can be achieved with an intervention strategy from the university authority by creating an enabling environment where the LGBTI could come out freely to access programmes targeted at the prevention and control of HIV/AIDS. The outcome of the study would also provide a platform to address the issue of accessing resources for LGBTI-targeted prevention and care for HIV/AIDS and the development and implementation of evidence-based interventions.

## Materials and methods

### Study design

This is a mixed method study that combined quantitative and qualitative approaches to collect data. The choice of this method places emphasises on the need to improve on monomethod research. However, only quantitative results are discussed for the purpose of this article.

#### Study site

The study site comprises the four campuses of Walter Sisulu University (Mthatha, Ibika, Buffalo City and Queenstown)-all located in the Eastern Cape of South Africa. The student population of over 21,000 comprises predominantly black South Africans with few coloured indigenes and foreign students. Considered a rural university, the main campus of Walter Sisulu University (Mthatha campus) and Ibika are located in the rural settings, while Buffalo City and Queenstown campuses area situated in urban areas with some sites in satellite towns.

#### Population and sample size

The target population of this study is the students of Walter Sisulu University. Sample size that comprised 3048 participants (1285 males and 1763 females) aged 17->38 years was purposively selected from the four campuses of Walter Sisulu University (Mthatha, Ibika, Buffalo City and Queenstown). The sample size from each campus was selected from different departments as follows: Mthatha campus 1300 (500 second level, 600 third level and 200 honours students); Ibika campus 348 (200 second level and 148 third level students); Buffalo City campus 800 (500 second level and 300 third level) and Queenstown campus 700 (400 second level and 300 third level). The total population of the students in this institution is 21,000

#### Data collection tool

Structured questionnaire comprising open and closed-ended questions with pre-coded answering categories was used for data collection. The questions from the questionnaire were partly adapted from the literature reviewed but largely from the authors understanding of the subject matter. The questionnaire was divided into four sections. Section A focuses on the socio-demographic profiles (age, sex, level of study, locality) of the participants. Section B solicits participants’ information concerning violence (corrective rape, physical attacks, coercive sex and other homophobic hostilities) against members of LGBTI, section C dwells on abuse (verbal insults-including derogatory remarks, bullying and name-calling), while section D focuses on discrimination (non-acceptance, denial of rights, prejudice and disapproval). For the purpose of this study, the pre-coded answering categories of ‘Agree’ and ‘Disagree’ are self-explanatory while ‘Decline’ indicate when a participant decided not to comment on the question. The questionnaire was pilot-tested on 136 students at the Nelson Mandela Drive delivery site of Mthatha Campus, Walter Sisulu University prior to the commencement of the study. This was done to validate the instrument of data collection.

#### Data collection procedure

The nature and aim of the study was explained to the participants. Only students who had agreed and signed the informed consent forms were given the questionnaire. Data were collected in all the four campuses of the institution (which are the only campuses of Walter Sisulu University) in the lecture halls where second level, third level and honours degree students were scheduled to have their lectures. The reason behind collecting data at all the campuses is to ascertain the views of participants representing the entire university students’ population. Data were collected between February and May 2015. Apart from the first year students, the questionnaire was administered to the students in other levels by the lead researcher and were collected at the spot after completion. Given that the questionnaire were administered when the students were scheduled to have their lectures, students were allowed the first 35 minutes of the lecture hour to complete the questionnaire. The exclusion of the first year students is not unconnected with the fact that, been registered new at the university, the first year students may not have in-depth knowledge of the subject matter. This is an attempt to reduce bias in the results, given that the study has to do with exploration of knowledge of university students on a specific subject matter occurring on all the campuses of the university. Out of the 3048 questionnaires administered, 155 were excluded because they had incomplete information. Therefore, 2893 were used in the final analysis.

#### Ethical issues

The Ethical committee of the Walter Sisulu University (WSU) approved the study protocol; and issued ethical clearance certificate [DVC (AA&R)/ DRD/SREC 09]. Informed consent was obtained from all participants before administering the questionnaires.

#### Statistical analysis

Quantitative responses from the questionnaire were transferred onto excel spreadsheet by a statistician. For the purpose of this publication, thematic coding was not created since the authors were interested in quantitative analysis. Data were analysed descriptively (percentages) using Statistical Package for Social Sciences (SPSS) version 21.0.

## Results

The knowledge and views of the participants concerning violence against the LGBTI community are presented in Table 1. The majority (47.7%) of the participants disagreed that it is justifiable to mete violence against members of the LGBTI community. However, 25.5% maintained an uncaring attitude if violence is perpetrated to the members of the LGBTI community.

**Table 1.** Participants’ views concerning violence against LGBTI community at WSU campuses.

Statements	Agree *n* (%)	Disagree *n*(%)	Decline *n*(%)
It is justifiable to be violent against members of LGBTI community	191 (6.6)	1380 (47.7)	1322 (45.7)
I would not care if violence is meted to members of LGBTI community	738 (25.5)	90 (3.1)	2066 (71.4)
The university should condemn violence against the LGBTI community I am aware of rape of lesbians on campus	1204 (41.6)	891 (30.8)	787 (27.2)
I have witnessed physical attacks on members of LGBTI community	579 (20.0)	1157 (40.0)	1157 (40.0)
I am aware of coercive sex with lesbians on campus	2040 (70.5)	289 (10.0)	564 (19.5)
	1082 (37.4)	1389 (48.0)	422 (14.6)

About 18.4% participants reported witnessing bullying of LGBTI community members on campus. Out of 2893 participants, 573 (19.8%) witnessed derogatory remarks targeting members of the LGBTI community on campus. About 32.4% reportedly witnessed verbal abuse against LGBTI community members, while 48.0% were aware of name-calling of members of the LGBTI in the university community (Table 2).

**Table 2.** Participants’ knowledge concerning abuse against members of the LGBTI community at WSU campuses.

Statements	Agree *n* (%)	Disagree *n* (%)	Decline *n* (%)
I am aware of verbal insults targeted at members of the LGBTI community on campus	937 (32.4)	1478 (51.1)	477 (16.5)
I have witnessed bullying of members of LGBTI on campus	532 (18.4)	0 (0.0)	2355 (81.4)
I am aware of derogatory remarks targeting LGBTI members	573 (19.8)	0 (0.0)	2320 (80.2)
I am aware of name-calling of members of the LGBTI on campus	1389 (48.0)	67 (2.3)	1438 (49.7)

Table 3 shows mixed reactions among participants concerning discrimination against LGBTI community members. While the majority of the participants declined to comment on how they feel about discrimination against the LGBTI community, few expressed anger at the discrimination. Majority of the participants also called for the university to put in place, programmes to address issues of discrimination against members of the LGBTI community.

**Table 3.** Participants’ views concerning discrimination against members of LGBTI community at WSU campuses.

Statements	Agree *n* (%)	Disagree *n* (%)	Decline *n* (%)
I have witnessed non-acceptance of members of LGBTI community on campus	981 (33.9)	738 (25.5)	1175 (40.6)
I am aware that some members of LGBTI community were denied their rights	327 (11.3)	246 (8.5)	2320 (80.2)
I am aware of prejudice as form of discrimination targeting LGBTI community members	943 (32.6)	833 (28.8)	1117 (38.6)
I have witnessed disapproval of act of homosexuals from students	1308 (45.2)	984 (34.0)	602 (20.8)
I am Angry about the discrimination against homosexuals	833 (28.8)	72 (2.5)	1987 (68.7)
I agree with discrimination against members of LGBTI community	579 (20.0)	90 (3.1)	2225 (76.9)
University to address issues of discrimination against members of LGBTI community	1721 (59.5)	631 (21.8)	541 (18.7)
University to have programmes to address discrimination Against members of LGBTI in the university community	1695 (58.6)	634 (21.9)	564 (19.5)

## Discussion

HIV/Acquired Immune Deficiency Syndrome (HIV/AIDS) is considered one of the most important global health issues due to the inability to cure the disease and its ability to spread across all geographical locations. The HIV/AIDS disease does not know boundaries, it does not respect race, religion or culture. The spread of HIV/AIDS is facilitated by global travel and population migration in addition to the link with increasing harmful drug use, changing lifestyles and sexual mores (Weinberg, 2005).

Among several factors responsible for transmission of HIV are lifestyles and unprotected sex. The LGBTI community is a high-risk group in relation to the virus HIV/AIDS and considered a bridge to HIV infection in the general population (Ainswoth et al., 2003). Control of HIV/AIDS among members of the LGBTI community is seen as a welcome development and it forms part of intervention strategies in global fight against the HIV/AIDS scourge.

Pervasive violent, abuse, harassment and discrimination being faced by some members of the LGBTI community in some regions of the world may be the reason behind unintended disclosure of sexual orientation by members of this group. However, where sexual orientation of members of the LGBTI community is not known, it denies members of the group the opportunity to benefit from tailored medical care designed for them. It is at the backdrop of invincibility of LGBTI community members that this study sought to ascertain factors responsible for unintended disclosure of their sexual orientation.

The findings of this study show that there is indeed harassment of members of the LGBTI community on all campuses of the Walter Sisulu University. Fact that 6.6% participants agreed, while 47.7% disagreed with justification of violence against members of LGBTI community on these campuses is a proof that there is indeed, harassment of this group within the university community (Table 1). Some studies done within university communities internationally and nationally support the findings of this study in many ways. A study conducted at a university community in USA to determine the incidence of discrimination, harassment and violence shows that nearly three-fourths experienced verbal abuse; 26% were threatened with violence; and 17% had personal property damaged. In another study, Evans and D’Augelli (1996) demonstrated that not all university campuses that have conducive and welcoming environments; that hostility towards lesbians and gay men is very common. Hatred towards members of the LGBTI community on South African university campuses have been documented. Ncanana and Ige (2014) revealed that students of Zululand University possess high levels of aversion toward homosexuals, though the level of ostensible acts of discrimination is low. The findings of Ncanana and Ige (2014) strongly agree with the findings of this study with regard to hatred directed to the members of the LGBTI community. The mixed feelings among students of Walter Sisulu University regarding the existence of LGBTI community, and the violence, abuse and discrimination meted against the group is of concern. While 1380 (47.7%) participants considered violence targeting members of the LGBTI community as wrong, 25% did not care if violence is meted against the group (Table 1). The lack of care regarding violence against members of the LGBTI community is a demonstration of indirect aversion by students of this university towards the group. But violence against members of the LGBTI community is a key factor militating against the control of HIV/AIDS among the group. It is thereby viewed as one of the many reasons why members of the LGBTI community decline to disclose their sexual orientation. The control of HIV/AIDS, sexually transmitted diseases (STDs), sexually transmitted infections (STIs) and order disorders associated with stigmatisation among the LGBTI community is a herculean task. This is because many members of this sexually minority group do avoid or delay care or receive care that is either inappropriate or of low quality. The decision to avoid or delay seeking care is informed by either negative personal experience or/and the assumption or expectation of negative experience based on the knowledge that other LGBTI people experienced such homophobic attacks. Violence also has an impact on mental health. There are much higher numbers of attempted suicide and self-harm across the LGBTI community when compared with the general community.

Abuse elicits among many other actions, low levels of confidence in the sensitivity and effectiveness of the school authorities to enforce security and safety of members of the LGBTI in this university community. Also, among same-sex attracted youth, the experience of verbal abuse doubled the likelihood of self-harm, and the experience of physical abuse tripled the likelihood of self-harm (Hillier, Turner, & Mitchell, 2005). This constitutes one of the factors that form barriers faced by members of the LGBTI community when it comes to seeking help that are unique to their sexual orientation and gender identity. These barriers, in turn, impact negatively on the effectiveness of programmes and services designed to assist members of the LGBTI community. In this university community, abuse, which is considered by members of the LGBTI community as one of the dangers of ‘outing’ oneself when seeking help, becomes one of the key factors associated with non-disclosure of one’s sexual orientation. The 32.4% and 48.0% participants, who witnessed verbal insults and other forms of name-calling of members of LGBTI sector, respectively, point to the fact that indeed acts of homosexuality do exist in this community (Table 2). Consequently, students’ attitudes to members of this sexually minority group, which cut across all forms of abuse, do not only stigmatise LGBTI members, they contribute significantly to non-disclosure of one’s sexual orientation (as belonging to the LGBTI community). Unrelenting verbal attacks on members of LGBTI community could create a hostile climate that is unbearable for them. It can undermine students ‘ability to focus at school. Therefore, the disclosure of one being a member of the LGBTI community becomes limited in most cases. It is this negative attitude towards the LGBTI community which limits the disclosure of homosexuality that places members at a disadvantage of accessing health programmes targeting the group despite their various health care needs. Cases of hostilities directed towards members of the LGBTI community as recorded in this study dates back in history. These hostilities directed towards members of the LGBTI within the university community as demonstrated in this study may be happening now, studies (Peters, 2003) done more than a decade ago showed that verbal and behavioural hostility directed towards lesbians and gay men do exist. Though these studies may be conducted outside the university settings, the findings concur with the findings of this present study. The negative impact of these hostilities is felt greatly in the health sector.

Internationally, there are concerns on the public health implications of different sexual behaviour. This is important in order to understand and tackle the spread of HIV/AIDS and other STDs (Misra & Chandiramani, 2005). As an entity of a larger community, the well-being of the LGBTI community is very pertinent, therefore elimination of the health disparities and enhanced efforts to improve on their health goes a long way to benefit the larger population. This is also necessary because it ensures that LGBTI individuals can lead long, healthy lives. In addressing the health concerns of the LGBTI community, the society stands to benefit in many ways. These include reductions in disease transmission and progression, increased mental and physical well-being, reduced health care costs and increased longevity among members. The findings of this study indicated that the LGBTI community faces societal stigma challenges. This societal stigma is also noticed in the findings of Centres for Disease Control and Prevention (CDC, 2011), where it is reported that ‘homophobia, stigma, and discrimination are social determinants of health that can affect physical and mental health; whether MSM seek and are able to obtain health services, and the quality of the services they receive’. The findings of Abaver, Cishe, Twaise, and Goon (2014), which showed that fewer members of the university community would like to be identified as belonging to the LGBTI community to avoid stigmatisation, also concur with this study. These challenges form barriers to health provisions to members of the LGBTI community, such barriers to health need to be addressed at different levels of society; such as health care settings, work places and schools. This becomes very important in order to increase opportunities for improving the health of MSM (CDC, 2011) and members of the LGBTI community at large.It is common knowledge that HIV-related stigma occurs among the general population. However, stigmatisation of HIV-positive individuals also occurs specifically within communities of gay men (Cloete, Simbayi, Kalichman, Strebel, & Henda, 2008; Dowshen, Binns, & Garofalo, 2009). Botnick (2000a) documented a growing division whereby HIV-negative gay men associate mainly or exclusively with other HIV-negative gay men, and vice versa. This has been linked with observations that HIV-positive gay men have an increased tendency to withdraw from both their usual social scenes and wider society (Botnick, 2000b). As a result, polarisation therefore exists: the resultant effect being negative impact on relationships and other aspects of physical and emotional health, social life, HIV testing behaviour, disclosure, disease prevention, and medication and therapy adherence (Botnick, 2000a; UNAIDS, 2009).

The complex and diverse history regarding the rights of LGBTI in South Africa shows that the legal and social status of this group have been influenced by so many factors which led to its abolition. Though South Africa’s post-apartheid constitution outlawed discrimination based on sexual orientation and rolled out several intervention strategies for the LGBTI community (Diale, 2014), societal acceptance of the group is generally lacking. Findings of Diale (2014) regarding level of acceptance of the LGBTI community members concurs with the findings of this study. This lack of general acceptance of this sexually minority group resurfaces in this study (Table 3). Of concern is the fact that 33.9% participants agreed having witnessed non-acceptance of members of LGBTI community on campus. This finding, basically from a population of rural-based tertiary institution however, contradicts the fairly acceptance of individuals who belong to LGBTI communities in major urban areas, such as Johannesburg, Pretoria, Durban and Cape Town (Johannesburg Gay Life, 2013). Health disparities of LGBTI people are linked to societal stigma, discrimination, and denial of their civil and human rights. Discrimination, which elicits series of stigma mechanisms (Earnshaw & Chaudoir, 2009), is viewed by members of the LGBTI as an unfair and unjust treatment to them based on their sexual orientation; the reason why adaptive radiation mechanisms which include non-disclosure of one’s sexual orientation are highly favoured to evade being stigmatised. Despite public education programmes and equal rights legislation, stigmatisation continues to be widespread, as seen from this study (Table 3), and can affect many aspects of life (Center for AIDS Prevention Studies [CAPS], 2006). Discrimination against members of the LGBTI community has been associated with, besides HIV/AIDS, high rates of psychiatric disorders (McLaughlin, Hatzenbuehler, & Keyes, 2010); substance abuse (Ibanez, Purcell, Stall, Parsons, & Gómez, 2005; Herek & Garnets, 2007) and suicide (Remafedi, French, Story, Resnick, & Blum, 1998). Coming from this sexually minority group which forms the integral part of the general population, such disorders coupled with the substance abuse and suicide tendencies put the larger population at both medical and societal risks. Discrimination comes in various forms: homophobic attitudes which results in non-acceptance; denial of basic right; and the general disapproval of homosexuality itself (Table 3). This view is supported by Herek (1995), who also found evidence of prejudice and discrimination against lesbians and gay men on campuses. High level of discrimination (32.6%) observed in this study (Table 3) contradicts the findings of Ncanana and Ige (2014) who revealed that, while students possess high levels of aversion toward members of the LGBTI community, the level of ostensible acts of discrimination is low.

Discrimination against members of LGBTI community-often hidden, under recorded and under reported-makes it difficult to gain an accurate representation of the extent of the medical issues facing members of this community. For instance, personal experience shows that lesbians who tell their health providers they are sexually active report being pressured to obtain birth control. This is because, the health provider who often equates female sexual activity with the possibility of pregnancy may not want to engaged in consulting a lesbian, given that this might be against his or her cultural or religious belief. Internalised homophobia results to wrong referrals by health providers. Gay men suffering from Human papilloma virus (HPV), who remain in the closet, may not be screened with anal Pap smear to detect early cancer. This increases the risk for HIV, since cancer is an opportunist infection. Self-denial regarding sexual orientation due to internalised homophobia is not uncommon among youths. These youths are denied access to accurate and comprehensive information about human sexuality and alternative lifestyles because of unrealistic fears of promoting homosexuality. The LGBTI people generally do experience poorer health, and there are no significant differences in terms of major health problems between them and the general population (Meads et al., 2009). Notwithstanding, issues affecting the LGBTI community include depression and anxiety, domestic violence, breast cancer (lesbians), HPV (gay men), mental health and suicide, as well as STDs and STIs. Identification of members of LGBTI community in this university is a huge challenge. However, consistent with Abaver and Cishe (2016) and Abaver et al. (2014), the participants in this present study witnessed violence (70.5%), abuse (48.0%) and discrimination (45.2%) against members of the LGBTI community. In their study, Abaver and Cishe (2016) identified members of the LGBTI as gay (5.6%), lesbian (7.9%) and bisexual (6.3%) at this same university (Walter Sisulu University). Similarly, Abaver et al. (2014) in their study concerning knowledge, perception and behaviour of students towards the LGBTI community at Walter Sisulu University, Eastern Cape, confirm the existence of gay (5.6%), lesbian (7.9%) and bisexual (6.3%) among the university community. It is assumed that these figures do not reflect the actual statistics of members of LGBTI in this community. This assumption emanates from the findings of this study where it is shown that various forms of violence, abuse and discrimination have been meted to different members of LGBTI community at different instances and campuses. The non-disclosure of sexual orientation by this group, therefore, is not surprising. This may not be unconnected with decades of history of pervasive violence and abuse targeted at this sexually minority group in South Africa. The difficulty associated with identifying members of the LGBTI community, however, became ameliorated with the outbreak of HIV/AIDS epidemic in South Africa which forced the LGBTI South Africans to reveal their sexual orientation, in order to be able to fight the disease and to ensure that those infected have access to life-saving medicines.

Discrimination like other factors that constitute stigmatisation, limits medical research and negatively impact the health care of LGBTI individuals. Lesbians bear the brunt of this aspect of health disparity most; a situation linked to what is considered to be a double minority status, and the oppression they experience for being both female and practicing homosexuality. Decriminalisation of homosexuality in this tertiary institution is advocated. The findings of this study which show that 1380 (47.7%) participants condemned violence directed at members of the LGBTI community should be the basis for this advocacy. The 32.4% participants who agree that they have witnessed insults targeting members of the LGBTI community on campus provides evidence for the university to initiate measures towards decriminalisation of homosexuality. The 45.2% who witnessed disapproval of act of homosexuality from students- all point to the fact that this particular university does not provide conducive learning environment for members of the LGBTI community. Hence the challenges faced in an attempt to control and prevent HIV transmission among this group considered to be at high risk of HIV/AIDS. The understanding of the extent to which members of LGBTI community are affected, the treatment they need, and the role they play in the overall transmission of HIV/AIDS epidemic in South Africa is very paramount. Without these, advocacy for resources to mitigate LGBTI-targeted prevention and care would not be feasible.

## Limitations

It should be noted that this study was designed to evaluate violence, abuse and discrimination as factors that constitute part of the reasons why LGBTI community members hide their identity. The study considered these factors in relation to overall resultant effect, which is stigma. The general survey did not place emphasis on individual key factor (violence, abuse and discrimination) but the overall negative impact these factors exert on the psychological behaviour of this key population that resulted to stigmatisation. As such the findings of this study should be interpreted bearing this in mind. Future studies should endeavour to elucidate more on abuse as key factor independent of violence.

Another limitation of the study is the inability to identify and screen members of LGBTI for incidence and prevalence of HIV. The instrument of data collection (the questionnaire) used in this study did not emphasise on sexuality as a factor in data collection. This should also be considered in future research.

## Conclusion

Despite legislations prohibiting all forms of violence, abuse and discrimination based on sexual orientation in South Africa, general acceptance of homosexuality by the larger community is low. This leads to members of the LGBTI community very unwilling to disclose their identity. Non-disclosure of identity by individuals of this group denies members the opportunity of accessing specific health care needs tailored at the prevention and control of STIs, such as HIV/AIDS. This calls for sensitisation across our universities in particular, and the larger society on the need to recognise and respect the rights of individuals irrespective of sexual orientation; and seek to modify attitudes of the larger population in order to ensure that people of different sexual orientations feel safe in our universities and the larger society (Oti-Boadi, Agbakpe, & Dziwornu, 2014). The inclusion of LGBTI issues in the university curriculum would help students address their own biases for better social interaction irrespective of sexual orientation; and is a step forward in the control and prevention of HIV/AIDS. This will provide enabling environment for people of different sexual orientations to freely identify themselves and access the specific healthcare needs that would eradicate the transmission of HIV/AIDS.

## Disclosure statement

No potential conflict of interest was reported by the author(s).
